# Supplementary material for: Resolving the full spectrum of human genome variation using Linked-Reads
Source: Genome Res. 2019 Apr;29(4):635–45. doi: 10.1101/gr.234443.118 (PMC6442396; doi:10.1101/gr.234443.118)
Supplement: Supplemental Material [file supp_gr.234443.118_Supplemental_longranger-2.2.zip › longranger-2.2.tar.bz2!/lr2.2-public/lib/stan/cmdstan/stan/doc/api/html/var__stack_8cpp_source.html]

Stan: src/stan/agrad/rev/var\_stack.cpp Source File


|  |  |
| --- | --- |
|  | Stan  2.5.0  probability, sampling & optimization |


- Main Page
- Related Pages
- Namespaces
- Classes
- Files

- File List
- File Members

All Classes Namespaces Files Functions Variables Typedefs Enumerations Enumerator Friends Macros Pages

- src
- stan
- agrad
- rev

var\_stack.cpp

Go to the documentation of this file.

1 #ifndef \_\_STAN\_\_AGRAD\_\_REV\_\_VAR\_STACK\_CPP\_\_

2 #define \_\_STAN\_\_AGRAD\_\_REV\_\_VAR\_STACK\_CPP\_\_

3

4 #include <stan/agrad/rev/var\_stack.hpp>

5

6 namespace stan {

7

8  namespace agrad {

9

10

11  // FIXME: manage all this in a thread-local singleton container

12

13  std::vector<chainable\*> var\_stack\_;

14  std::vector<chainable\*> var\_nochain\_stack\_;

15  std::vector<chainable\_alloc\*> var\_alloc\_stack\_;

16  memory::stack\_alloc memalloc\_;

17

18  std::vector<size\_t> nested\_var\_stack\_sizes\_;

19  std::vector<size\_t> nested\_var\_nochain\_stack\_sizes\_;

20  std::vector<size\_t> nested\_var\_alloc\_stack\_starts\_;

21  }

22

23 }

24

25 #endif

var\_stack.hpp

stan::agrad::memalloc\_

memory::stack\_alloc memalloc\_

**Definition:** var\_stack.cpp:16

stan::memory::stack\_alloc

An instance of this class provides a memory pool through which blocks of raw memory may be allocated ...

**Definition:** stack\_alloc.hpp:76

stan::agrad::nested\_var\_stack\_sizes\_

std::vector< size\_t > nested\_var\_stack\_sizes\_

**Definition:** var\_stack.cpp:18

stan::agrad::nested\_var\_alloc\_stack\_starts\_

std::vector< size\_t > nested\_var\_alloc\_stack\_starts\_

**Definition:** var\_stack.cpp:20

stan::agrad::var\_stack\_

std::vector< chainable \* > var\_stack\_

**Definition:** var\_stack.cpp:13

stan::agrad::var\_alloc\_stack\_

std::vector< chainable\_alloc \* > var\_alloc\_stack\_

**Definition:** var\_stack.cpp:15

stan::agrad::nested\_var\_nochain\_stack\_sizes\_

std::vector< size\_t > nested\_var\_nochain\_stack\_sizes\_

**Definition:** var\_stack.cpp:19

stan::agrad::var\_nochain\_stack\_

std::vector< chainable \* > var\_nochain\_stack\_

**Definition:** var\_stack.cpp:14


---

[ Stan Home Page ]

*© 2011–2014,
Stan Development Team.*
